# A Transcriptomic Approach to Search for Novel Phenotypic Regulators in McArdle Disease

**DOI:** 10.1371/journal.pone.0031718

**Published:** 2012-02-09

**Authors:** Gisela Nogales-Gadea, Inés Consuegra-García, Juan C. Rubio, Joaquin Arenas, Marc Cuadros, Yolanda Camara, Javier Torres-Torronteras, Carmen Fiuza-Luces, Alejandro Lucia, Miguel A. Martín, Elena García-Arumí, Antoni L. Andreu

**Affiliations:** 1 Departament de Patologia Mitocondrial i Neuromuscular, Hospital Universitari Vall d'Hebron, Institut de Recerca (VHIR), Universitat Autónoma de Barcelona, Barcelona, Spain; 2 Experimental Neurology Laboratory, Institut de Recerca HSCSP, Universitat Autònoma de Barcelona, Barcelona, Spain; 3 Spanish Network for Research in Rare diseases (CIBERER), Instituto de Salud Carlos III, Spain Centro de Investigación, Hospital Universitario 12 de Octubre, Madrid, Spain; 4 Unidad de Proteómica, Instituto de Investigación, Hospital Universitario 12 de Octubre, Madrid, Spain; 5 Unidad de Genómica, Instituto de Investigación, Hospital Universitario 12 de Octubre, Madrid, Spain; 6 Universidad Europea de Madrid, Madrid, Spain; 7 Centro de Investigación, Hospital Universitario 12 de Octubre, Madrid, Spain; University of Rome, Italy

## Abstract

McArdle disease is caused by lack of glycogen phosphorylase (GP) activity in skeletal muscle. Patients experience exercise intolerance, presenting as early fatigue and contractures. In this study, we investigated the effects produced by a lack of GP on several genes and proteins of skeletal muscle in McArdle patients. Muscle tissue of 35 patients and 7 healthy controls were used to identify abnormalities in the patients' transcriptomic profile using low-density arrays. Gene expression was analyzed for the influence of variables such as sex and clinical severity. Differences in protein expression were studied by immunoblotting and 2D electrophoresis analysis, and protein complexes were examined by two-dimensional, blue native gel electrophoresis (BN-PAGE). A number of genes including those encoding acetyl-coA carboxylase beta, m-cadherin, calpain III, creatine kinase, glycogen synthase (GS), and sarcoplasmic reticulum calcium ATPase 1 (SERCA1), were found to be downregulated in patients. Specifically, compared to controls, GS and SERCA1 proteins were reduced by 50% and 75% respectively; also, unphosphorylated GS and SERCA1 were highly downregulated. On BN-PAGE analysis, GP was present with GS in two muscle protein complexes. Our findings revealed some issues that could be important in understanding the physiological consequences of McArdle disease: (i) SERCA1 downregulation in patients could result in impaired calcium transport in type II (fast-twitch) muscle fibers, leading to early fatigability during exercise tasks involving type II fibers (which mostly use glycolytic metabolism), i.e. isometric exercise, lifting weights or intense dynamic exercise (stair climbing, bicycling, walking at a very brisk pace), (ii) GP and GS were found together in two protein complexes, which suggests a new regulatory mechanism in the activity of these glycogen enzymes.

## Introduction

McArdle disease (glycogen storage disease type V) is an autosomal recessive disorder caused by a deficiency of muscle glycogen phosphorylase (GP), the enzyme that catalyzes the first step in glycogen catabolism [1], [2]. Because of this deficiency, daily life activities involving isometric exercise (e.g. lifting weights) or dynamic exercise (e.g. stair climbing) trigger exercise intolerance in McArdle patients, which manifests as early fatigue and contractures, sometimes accompanied by rhabdomyolysis and myoglobinuria [3]. Any muscle in the body can be affected and approximately one-third of the patients also develop proximal muscle fixed weakness and wasting with aging [4], [5].

The rationale for exercise intolerance in these patients has been traditionally explained through metabolic depletion. Blockade of glycogen breakdown compromises aerobic and anaerobic glycolysis [6]. The oxidative capacity of the patient's muscle is also impaired because of their ability to produce pyruvate - a molecule that plays an anaplerotic role in the Krebs cycle - is severely reduced [3]. Nonetheless, it is also of note that the extent of the oxidative defect is substrate dependent; i.e., it can be partially corrected by increasing the availability of alternative oxidative substrates (e.g. glucose) to working muscles [7]. The reduced potential for oxidative phosphorylation is reflected by marked reduction in the [ATP]/[ADP][P*i*] ratio during muscle contractions compared with healthy controls (as shown with phosphorus magnetic resonance spectroscopy) [8]. With regard to this, the premature muscle fatigue and cramping of McArdle patients is associated with an increased accumulation of Pi and probably ADP in skeletal muscle; accumulations of Pi and ADP are indeed known to inhibit (i) myofibrillar, (ii) sarcoplasmic reticulum (SR) Ca^2+^, and (iii) membrane Na^+^-K^+^-ATPase reactions

On the other hand, an additional hallmark of the disorder, which is not necessarily related to exercise intolerance is the chronically high subsarcolemmal and intermyofibrillar glycogen store deposits that are present in patients' muscles [9], [10]. Glycogen synthesis is catalyzed by the enzyme, glycogen synthase (GS), which exerts the opposite function of GP. Both enzymes share regulatory mechanisms: covalent modification, allosteric activation, and enzymatic translocation [11]. The role of GS in glycogen accumulation is not fully understood, and a decrease in GS activity has been reported in McArdle patients [10], [12]. The activity of enzymes regulating covalent modification of GS, glycogen synthase kinase and protein phosphatase 1, do not explain the reduction in GS activity present in the patients [12].

In this study, we characterized the transcriptomic profile of a selection of key genes involved in the maintenance of skeletal muscle function. We identified other chronic protein alterations that occur in the muscle of McArdle patients in addition to GP deficiency: (i) a near absence of the unphosphorylated SERCA1; and (ii) a reduction of GS, with a shift through the inactive forms of both proteins. Furthermore, protein studies suggested that GP binds with GS to form protein complexes, thereby creating an additional mechanism to regulate glycogen metabolism.

## Materials and Methods

### Subjects

35 Spanish McArdle patients (P1–P35), some of them previously characterized by molecular techniques [13], [14], [15], [16] were included in the study (Table 1). To avoid differences due to lower muscle training compared with non-patients, 7 healthy volunteers with sedentary life-style habits were used as controls (C1–C7). Age range was 17 to 71 years in the patient group and 23 to 50 years in the control group. Patients presented a broad representation of the different clinical severity groups, in accordance with a previously reported scale by Martinuzzi et al. [17]. Patients P1–P34 and controls C1–C5 were studied in the RNA analysis. Later, patient P35, and controls C6 and C7 were included in the studies of 2D and blue native gel electrophoresis, due to the high amount of protein needed for these analyses. Written consent was obtained from all participants. The study was approved by the Ethics Committee of *Hospital Universitario 12 de Octubre* and was performed in accordance with the Declaration of Helsinki for Human Research. A skeletal muscle biopsy was obtained from patients and controls.

**Table 1:** Demographic data and information obtained by low-density array analysis in patients and healthy controls.

Participant number	Sex	Age^a^	Muscle biopsy	GP activity^b^	Genotype^c^	Clinical severity scale^d^
P1	M	59	B	0	p.W798R/p.W798R	3
P2	M	18	NA	0	p.R194W/p.P797VfsX19	2
P3	M	34	NA	0	p.R50X/p.R94W	2
P4	M	19	NA	0	p.R50X/p.G205S	1
P5	M	22	B	0	p.R50X/p.G205S	2
P6	M	17	B	0	p.R50X/p.A660D	1
P7	M	13	B	0	p.R50X/p.W798R	3
P8	M	33	NA	0	p.R50X/p.W798R	0
P9	F	21	B	0	p.N134KfsX161/p.R491AfsX7	3
P10	M	18	Q	0	p.W388SfsX34/p.K754fsX49	2
P11	F	68	NA	0	p.R50X/p.L5vfsX22	0
P12	F	32	B	0	p.R50X/p.L5vfsX22	1
P13	F	63	NA	0	p.R50 X/p.Q73HfsX7	3
P14	F	40	B	0	p.R50X/p.534VfsX5	1
P15	M	NA	B	0	p.R50X/p.534VfsX5	1
P16	M	35	NA	0	p.R50X/p.K754NfsX49	2
P17	F	31	Q	0	p.R50X/p.R50X	2
P18	M	71	Q	0	p.R50X/p.R50X	0
P19	F	21	B	0	p.R50X/p.R50X	3
P20	M	52	NA	0	p.E125X/p.E125X	3
P21	F	56	Q	0	p.W798R/p.W798R	1
P22	F	20	Q	0	p.R50X/p.L354P	0
P23	F	51	Q	0	p.R50X/p.R50X	3
P24	M	51	B	0	p.R50X/PIM	3
P25	M	21	B	0	p.G205S/p.Q176_M177insVQ	2
P26	F	26	Q	10	PIM/PIM	2
P27	M	25	D	0	p. G174D/c.1827G>A	2
P28	M	NA	NA	0	p.R50X/c.1969+214_2177+369del	2
P29	F	47	Q	0	p.R50X/p.R602W	3
P30	F	29	NA	ND	p.R50X/p.R50X	1
P31	M	51	NA	ND	p.R771PfsX33/p.R771PfsX33	1
P32	F	28	NA	ND	p.L5VfsX22/p.K754NfsX49	2
P33	M	61	Q	0	p.G205S/p.G205S	1
P34	M	23	NA	ND	p.R50X/p.A660D	1
P35	M	21	NA	0	p.R50X/p.R50X	3
C1	F	28	Q			
C2	F	35	Q			
C3	F	23	B			
C4	M	27	B			
C5	M	43	B			
C6	F	50	B			
C7	F	42	B			

PIM, possible intronic mutation.

a Age at the time the skeletal muscle biopsy was collected.

b GP activity units are µmol/min/g tissue.

c Mutations identified in the protein translation product are indicated by a “p” and in the transcript level by a “c” (www.hgvs.org/mutnomen). GenBank reference sequences were NP_005600.1 and NM_005609.1.

d Patients's clinical severity was classified based on a numerical scale following the criteria defined by Martinuzzi et al. [17]

Abbreviations: M, male; F, female; NA, data non available; B, biceps; Q, quadriceps; D, deltoid; ND, not determined;

### Low Density Array (LDA) and Real-Time PCR analysis

RNA samples were obtained as previously described [13]. Customized low-density arrays (LDAs) were configured to study 48 genes (see Supplementary Table S1) using TaqMan assays (Applied Biosystems, Foster City, CA, USA). The genes we studied play an important role in major muscle functions: metabolism, membrane transport, mitochondrial function/biogenesis and dynamics, maintenance of cytoskeleton, contractile function, calcium reuptake in sarcoplasmic reticulum, control of muscle growth, atrophy and differentiation, neuromucular transmission, anti-stress protection, inflammation and cell death.

Samples were run in duplicate on the ABI 7900HT system (Applied Biosystems). The PCR conditions were 2 min at 50°C and 10 min at 94.5°C, followed by 50 cycles of two steps, 30-s at 97°C and 1 min at 59.7°C. Cyclophilin A (*PPIA*) was used for data normalization, the calibrator sample was C2, and the software for the analysis was SDS 2.3 and RQ 1.2 (Applied biosystems). The muscle GP (*PYGM*) (Hs00194493_m1) and leptin (*LEP*) (Hs00174877_m1) genes were quantified by real-time PCR using TaqMan fluorogenic probes, as described elsewhere [13].

### Immunoblot Analysis

Skeletal muscle specimens were homogenized in 40 mM β-glycerophosphate, 40 mM NaF, 10 mM EDTA, and 20 mM mercaptoethanol (pH 6.8). Proteins were resolved on 10% SDS polyacrylamide gel. Antibodies against SERCA1, calpain 3, muscle cadherin (m-cadherin) (Abcam, Cambrige, UK), muscle GS (Cell signaling, Danvers, USA), and muscle GP (primary antibody generated in Dr. Martinuzzi's laboratory) were utilized. The horseradish peroxidase-conjugated secondary antibodies included goat anti-mouse, goat anti-rabbit (Jackson Laboratories, PA, USA), and donkey anti-goat (Santa Cruz, Santa Cruz, CA, USA). Membranes were developed with ECL (Amersham, Buckinghamshire, UK). Images were obtained with a LAS-3000 system (FujiFilm, Tokyo, Japan) and quantified with NIH image J (version 1.37) software (Scion image, NIH). Cyclophilin A (Enzo Life Sciences, Plymouth Meeting, PA, USA) was used as the housekeeping protein.

### 2D electrophoresis Analysis

Skeletal muscle was homogenized in lysis buffer (10 mM Tris pH 8.0, 1 mM EDTA, 150 mM NaCl, 10 µg/mL leupeptin, 10 µg/mL aprotinin, 10 µg/mL pepstatin A, and 0.5 mM PMSF). Seventy micrograms of protein were applied by cup loading and focused using the IPGphor 3 electrophoresis unit (GE Healthcare, Waukesha, WI, USA). For the second dimension, IPG strips were reduced for 15 min in SDS-equilibration buffer (6 M urea, 50 mM Tris-HCl, pH 8.8, 20% glycerol, 10% SDS, 1% w/v DTT, and a trace of bromophenol blue); proteins were then alkylated in the same buffer containing 2.5% w/v iodoacetamide. Second dimension separation was performed on 10% gel. The same primary antibodies were used, with the exception of muscle GP (Abcam, Cambrige, UK); α-tubulin (Sigma, Saint Louis, USA) was used for data normalization.

### Blue Native Gel Electrophoresis (BN-PAGE)

BN-PAGE [18] was performed with 3% to 12% polyacrylamide gradient gels. Proteins were extracted with 1% *n*-dodecyl-β-D-maltoside and diluted with a buffer containing 1.5 M aminocaproic acid, 2 mM EDTA, and 50 mM bis-Tris (pH 7.0). Serva Blue G was added to solubilize proteins to a final concentration of 0.01 mg/mg protein and 50 µg of protein were loaded into each lane. Denatured proteins were resolved in a second dimension and the following steps were the same as those used in 2D analysis.

### Statistical analysis

Differences in LDA gene expression were evaluated with a permutation analysis and *p*-values were adjusted for multiple comparisons using the False Discovery Rate control method [19]. The influence of other variables on gene expression was tested. Each gene was adjusted to different linear models and all possible variable combinations were tested. The model for qualitative variables (sex, muscle biopsy, Martinuzzi index) was equivalent to analysis of variance, and the model for numeric and quantitative variables (age, amount of *PYGM* and *LEP* transcripts) was equivalent to analysis of covariance. The statistical analysis for immunoblotting was performed using SPSS (SPSS15.0, Chicago, IL, USA). Differences between groups were analyzed with the non-parametric Mann Whitney *U* test.

## Results

### Expression of ACACB, CAPN3, CADH15, CKMM, GYS1 and SERCA1 is down-regulated in McArdle patients

**Figure 1:**
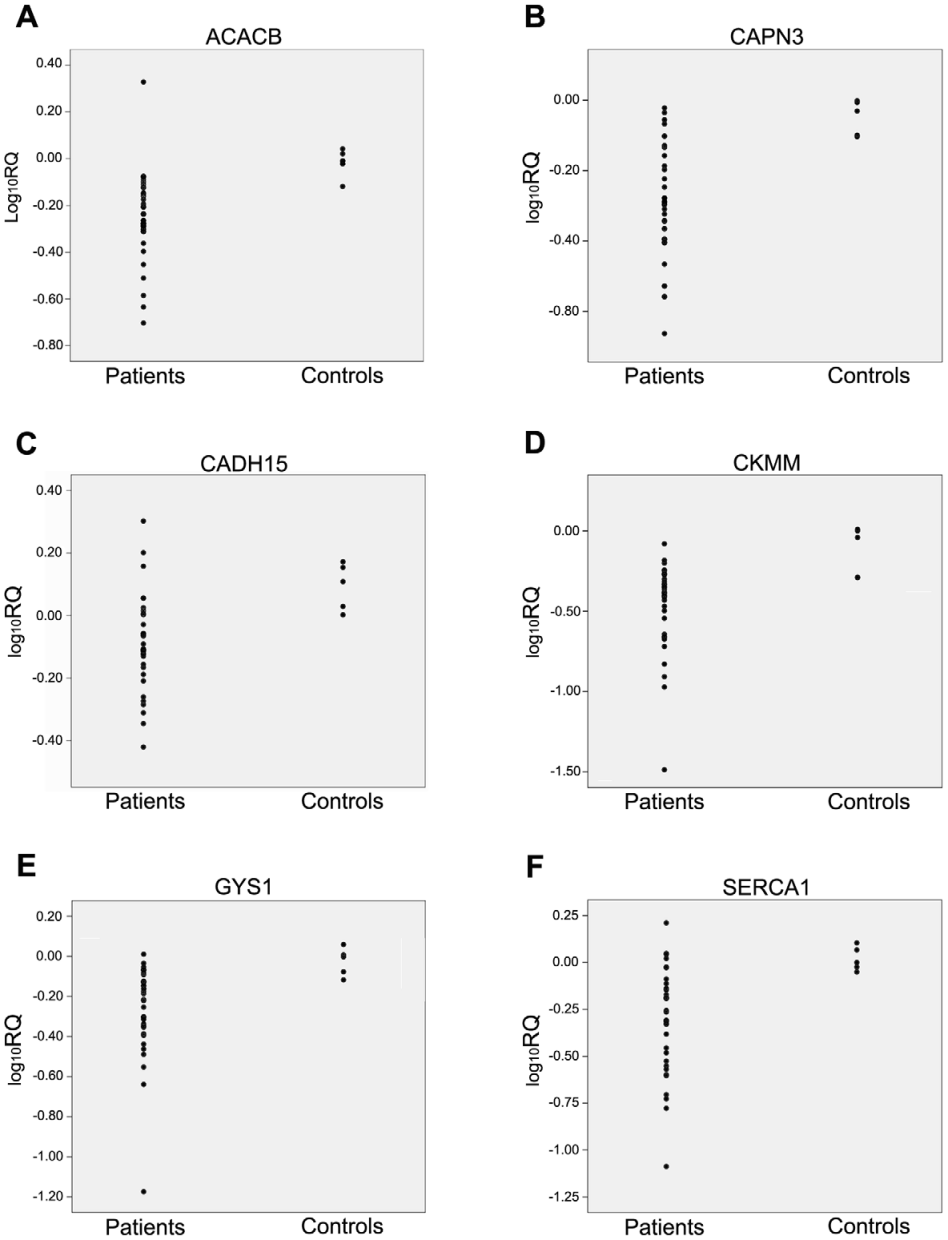
Scatter-plots for the six genes presenting the highest differential expression between patients and controls (*p*<0.01). A. Acetyl-CoA carboxylase beta (*ACACB*); B. Calpain III large subunit (*CAPN3*); C. M-cadherin (*CADH15*); D. Muscle creatine kinase (*CKMM*); E. Muscle glycogen synthase (*GYS1*); F. Sarcoplasmic reticulum Ca^2+^ ATPase 1 (*SERCA1*). Each subject is represented by a dot.

Expression analyses were performed on skeletal muscle from 34 patients (P1–P34) and 5 healthy individuals (C1–C5) (17 women and 22 men). Statistically significant differences between patients and controls (*p*<0.01) were observed for 6 of the studies genes studied (Figure 1A–1F): *ACACB*, *CAPN3*, *CADH15*, *CKMM*, *GYS1* and *SERCA1*. Patients presented a lower amount of transcripts than controls, for all of the 6 aforementioned genes. Additionally, 10 other genes showed significantly decreased expression in patients compared with controls (*p*<0.05) (Supplementary Table S2).

The individuals included in the study group showed considerable heterogeneity for certain characteristics (Table 1). Therefore, the data were reanalyzed to exclude these factors as the cause of the differential gene expression, and no associations were identified for sex, age, and muscle type. *PYGM* genotype was analyzed by classifying the patients in three groups: (i) two missense mutations, (ii) one missense mutation and one nonsense mutation, or (iii) two nonsense mutations. The expression profile of these genes was not influenced by the patients' PYGM genotype. On the other hand, to determine whether the amount of adipose tissue in skeletal muscle could influence the gene expression pattern, leptin (*LEP*) expression [20] was studied, and no differences in content of *LEP* transcripts were found. This lack of correlations, suggests that the differences in the expression pattern was solely due to the existence of the enzyme (GP) deficiency.

### SERCA1 and muscle GS protein content were reduced in patients

**Figure 2:**
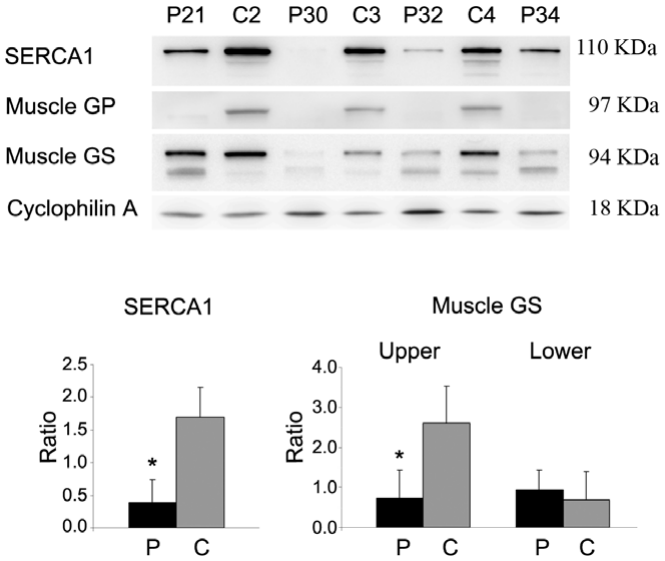
Immunoblotting of patients and controls for SERCA1, muscle GP, muscle GS, and cyclophilin A proteins. Upper panel: immunobloting for the indicated proteins; Lower panel: densitometric analysis for SERCA1 and muscle GS immunodetection. Intensity ratios of protein bands to cyclophilin A are shown as mean±SD. Symbol: * *p*<0.05; Upper, upper band of muscle GS immunoblotting; Lower, lower band of muscle GS immunoblotting; Patients P21, P30, P32 and P34; Controls C2, C3, C4. P: Patients; C: Controls.

We studied the protein products from four of the genes identified to be differentially expressed in patients. These included *SERCA1*, *GYS1*, *CAPN3* and *CADH15*. Immunoblots were performed on 4 patients (P21, P30, P32 and P34) and 3 controls (C2, C3, and C4). SERCA1 and glycogen synthase (GS) protein levels were markedly decreased (showing a 75% and 50% reduction respectively) in patients respect to controls (*p*<0.05) (Figure 2). The upper GS band, corresponding to its expected weight of 94 KDa, was even more markedly decreased, with a 72% reduction in patients. In addition, some lower GS bands appeared, suggesting changes in the protein phosphorylation rate.

### Patients presented reduced unphosphorylated muscle GS and a near absence of unphosphorylated SERCA1

**Figure 3:**
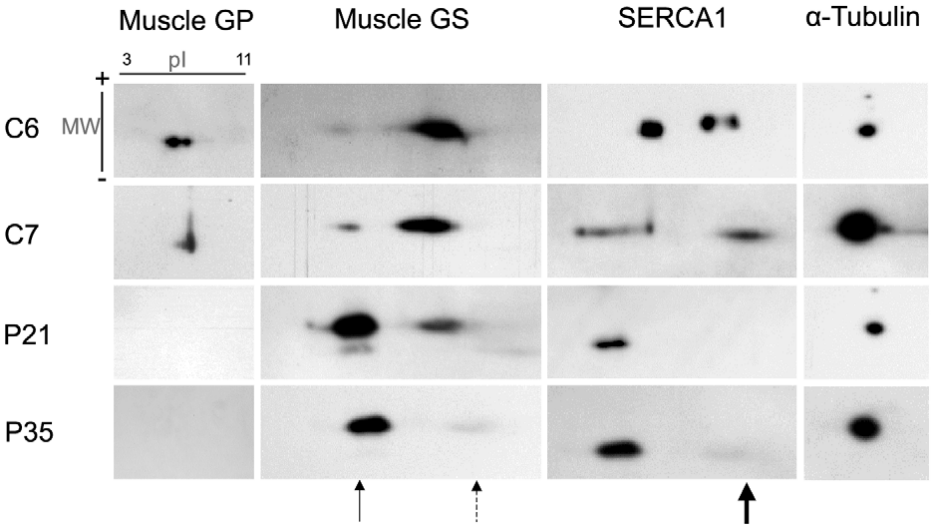
2D gel analysis of muscle GP, muscle GS, SERCA,1 and α-tubulin in 2 controls and 2 patients. In the first dimension, isoelectric focusing separated proteins by pH 3–11. In the second dimension, proteins were separated by molecular weight. Abbreviations: pI, isoelectric point; MW, molecular weight. Patients P21, P35; Controls C6, C7. Thin arrow indicates acidic muscle GS, discontinuous arrow indicates basic muscle GS and thick arrow indicates basic SERCA1.

As in the immunoblot study, 2D gel analysis showed an absence of muscle GP in patients (Figure 3). GS appeared as two clear spots by 2D gel analysis (thin arrows), but the patients showed higher amounts of acidic muscle GS (continuous thin arrow), which corresponds to phosphorylated forms of muscle GS, whereas the controls showed a higher amount of more basic muscle GS (discontinuous thin arrow), corresponding to unphosphorylated forms. Compared to the controls, patients showed a 20% reduction in unphosphorylated GS and a 60% increase in phosphorylated GS. Similarly, SERCA1 protein presented as two differentiated spots, but the spot corresponding to basic (unphosphorylated) SERCA1 was almost absent (thick arrow) in patients.

### GP formed two protein complexes with GS, which were lower in McArdle patients

**Figure 4:**
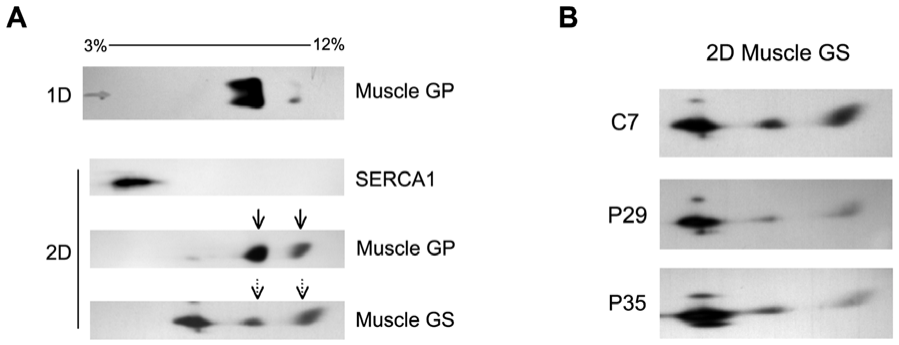
BN-PAGE of muscle GP, muscle GS, and SERCA1. A. BN-PAGE for control C4. In the first dimension (1D), acrylamide percentage ranges from 3% to 12%. In the second dimension (2D), continuous arrows indicate the 2 protein complexes in which muscle GP participates. Discontinuous arrows show muscle GS forming complexes with muscle GP. B. Second dimension of muscle GP BN-PAGE in a control (C7) and patients (P29 and P35).

BN-PAGE performed on a control sample (Figure 4A) showed 2 spots (continuous arrows) for muscle GP: a spot corresponding to a complex running in a medium acrylamide concentration, and a spot corresponding to a second complex, in a lower acrylamide concentration, suggesting the presence of muscle GP in different protein complexes. The composition of these complexes was analysed by a second dimention electrophoresis (SDS-PAGE) and subsequent immunoblotting. SERCA1 immunodetection revealed no overlapping of the protein with muscle GP, suggesting no interaction between both proteins. However, when the protein complexes were analysed for the presence of muscle GS, two of the three complexes previously detected for muscle GP in the first dimension, overlapped with the signal for GS protein (discontinuous arrows), suggesting that both GS and GP are present in these complexes. In patients P29 and P35, no signal was detected for muscle GP in comparison with the control (C7) (data not shown). When testing for muscle GS complexes in these patients, the same complexes as those observed in controls were present, but the two complexes normally containing muscle GP, showed a 70% reduction (Figure 4B).

## Discussion

This study evidences the potential of transcriptomic arrays combined with proteomic analysis to identify proteins involved in the pathophysiology of metabolic disorders affecting the skeletal muscle. Given that sample size requirements for RNA studies are much lower than those for protein analysis approaches, we first performed tests on the expression profile of muscle transcripts with the aim of identifying those genes whose expression was significantly differential in patients. Amongst the 16 genes whose transcripts levels were found to be significantly lower compared with controls, we found both *GYS1* and *SERCA1* genes which we decided to analyse at their protein level due to their well-known key role in muscle function.

Skeletal muscle is the major site of insulin-stimulated glucose uptake, and most of this glucose is stored as glycogen through GS activity [21]. Evidence of deregulation in this pathway has been reported in McArdle patients, who have decreased insulin action in skeletal muscle compared with healthy controls after a hyperinsulinemic clamp, resulting in lower glucose use and lower GS activity [10]. McArdle patients have also been shown to have low GS activity during exercise [12]. Immunoblotting results (see Figure 2) are similar to those obtained in a previous study with McArdle patients, both showing a decrease in GS protein compared with healthy controls [10]. We also could identify several bands that represent different phophorylated forms of the enzyme. GS has 9 residues that can be phosphorylated, causing progressive inactivation of the enzyme [21]. With the use of 2D gels, we showed that, compared with controls, patients had an increase of inactive GS and a decrease of active forms. Some authors have shown that high glycogen in skeletal muscle causes lower insulin-stimulated glycogen synthesis and decreased glycogen synthase activation [22]. Therefore the high glycogen accumulation in muscle biopsies of these patients could be one of the contributors to GS inactivation.

BN-PAGE showed that GP forms two complexes in which GS is also present. This is relevant as some alterations in GS activity and phosphorylation occurring in McArdle patients cannot be fully explained by the decrease of enzyme activities responsible for the covalent modification of the enzyme [12]. The other two control mechanisms involved are allosteric regulation and enzyme translocation [11], but neither explains the low phosphorylation rate of muscle GS in these patients. The presence of muscle GP-GS complexes suggest that their common allosteric regulators and covalent modifiers function better if both proteins are in physical vicinity. For example, the onset of contraction results in a release of Ca^2+^ stores from the SR, which activates phosphorylase kinase; this in turn, activates GP and inactivates GS, both by phosphorylation. Protein complexes containing these two enzymes are expected to allow more flexible, efficient, and faster coupling between synthesis and degradation of glycogen [23]. The presence of GP in the complex also seems to stimulate GS binding, as evidenced by the fact that McArdle patients presented 30% less GS in comparison with healthy controls.

A decreased rate of Ca^2+^ reuptake by the SR (through SERCA pumps) can cause slowing of relaxation and contribute to decreased muscle force production in fatigued muscles. Indeed, muscle fatigue is usually accompanied by a slowing of relaxation and high rates of glycolytic ATP production, since up to 80% of the ATP consumed during contraction is required for optimum muscle relaxation [24], [25]. Thus, the decrease of SERCA1 protein we found in our patients compared with controls is of particular interest. Partial reductions in the activity [26] or total amount of SERCA1 due to *SERCA1* mutations cause a disorder known as Brody myopathy [26], a rare skeletal muscle condition that shares some common clinical features with McArdle disease, i.e.patients experience progressive muscle stiffness during exercise, leading to cramps [27]. Lack of SERCA1 also causes neonatal death in mice [28] SERCA1 is a Ca^2+^transporter ATPase located in the SR of fast twitch, type II fibers. Thus, SERCA1 is essential for normal development of contraction-relaxation cycles in these fibers, which are mainly glycogenolytic and therefore more sensitive to GP deficiency. Thus, SERCA1 reduction may preferentially affect those exercise tasks involving type II muscle fibers, i.e. isometric exercise, lifting weights or intense dynamic exercise (stair climbing, running, walking at very brisk pace, bicycling). This hypothesis is indirectly supported by the results of a recent study by our group using electromyography, in which McArdle patients showed excessive fiber recruitment during dynamic (cycle-ergometry) exercise, a phenomenon which was more marked at high intensities [29]. Interestingly, the low SERCA1 levels found in 2D gels in our McArdle patients is similar to that reported for Brody patients [30], yet McArdle patients showed a preferential reduction in SERCA1 unphosphorylated forms. Cuenda et al [30] found that GP inactive status affects SERCA, producing a shift towards its ATP binding conformation. Additionally, concentrations of calcium, ATP, ADP, and Pi, as well as pH values [31] can modify the phosphorylation rate in SERCA1, and some of these factors are reported to be deregulated in McArdle disease, notably increased [ADP] and [Pi] [4].

The biochemical bases of muscle fatigue in McArdle disease are not fully understood and a reduction in sodium-phosphate ATPases has been described as a possible cause of fatigability in McArdle disease [32]. As mentioned above, SERCA1 downregulation may also participate in the early fatigue that McArdle patients experience during exercise [3]. The reduction in both ATPases could also be related, since a preferential glycogenolytic energy supply and close membrane localization has been proposed to exist between the glycolytic enzymes and these channels [33]. Although SERCA1 is not present in muscle GP complexes, some reports provided evidence that they are located in close proximity. Meyer and coworkers [34] isolated protein-glycogen complexes and found glycogen particles and enzymes related with glycogen metabolism, among them GP, in the light fraction, and elements of the SR characterized by strong ATPase activity in the heavy fraction. Other authors reported the presence of SR in the sarcomeric I band, where glycogen deposits are located, and where glycogen is markedly reduced during muscle activity [35]. Moreover, calcium uptake in the SR can be supported solely through enzymatic breakdown of glycogen by GP [35], [36].

**Figure 5:**
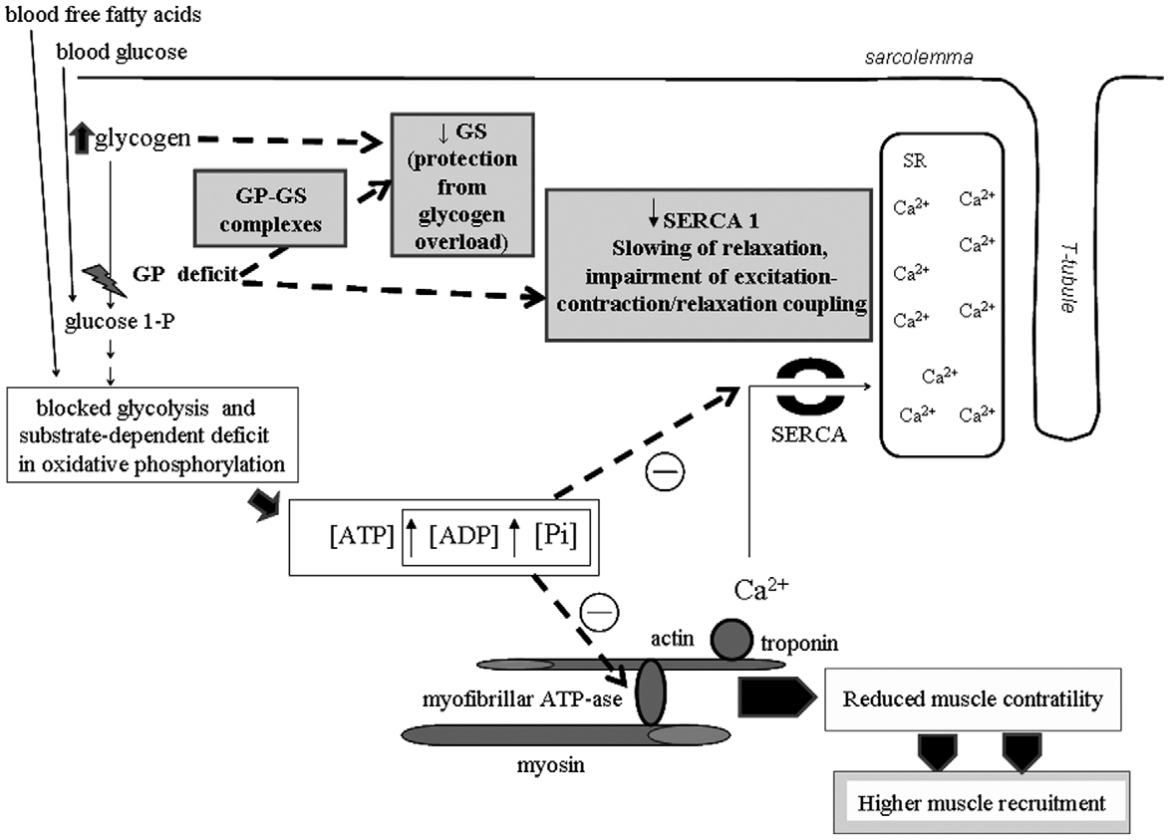
Schematic representation of the main findings and conclusions (in grey squares) of our study with regard to the pathophysiology of McArdle disease. Abbreviations: GS (glycogen synthase), GP [glycogen phosphorylase, muscle isoform (myophosphorylase)], SERCA1 (sarcoplasmic reticulum Ca^2+^-ATPase 1).

The main findings of our study are schematized in Figure 5. In summary, our results suggest that the absence of GP in McArdle patients may result in down-regulation of two control points in the metabolism of skeletal muscles, i.e. (i) SERCA1 protein levels and phosphorylation and (ii) the amount of GS. SERCA1 downregulation in patients could result in impaired calcium transport in type II muscle fibers, leading to early fatigability. On the other hand, the muscle of McArdle patients shows a shift towards inactive GS forms, protecting these patients from the occurrence of muscle glycogen overload. Some contributors to GS inactivation can be the deregulation of protein complexes GP-GS and the high glycogen accumulation in skeletal muscle. Overall, this study shows the existence of a link between muscle GP, muscle contraction-relaxation, and glycogen synthesis that may have a primary role in the pathophysiological events occurring in this disorder.

## Supporting Information

Table S1 Genes studied in the customized low density array. The genes of study are classified in groups depending on their role in muscle function: 1 = metabolism; 2 = membrane transport; 3 = mitochondrial function/biogenesis and dynamics; 4 = maintainance of cytoskeleton; 5 = contractile function; 6 = control of muscle growth; 7 = neuromuscular transmission; 8 = anti-stress protection; 9 = inflammation; 10 = cell death; 11 = calcium reuptake in sarcoplasmic reticulum; 12 = housekeeping gene.
(DOC)

Table S2 Genes with statistically significant differential expression (p<0.05) between patients and controls.
(DOC)
