# Supplementary material for: A Transcriptomic Approach to Search for Novel Phenotypic Regulators in McArdle Disease
Source: PLoS One. 2012 Feb 9;7(2):e31718. doi: 10.1371/journal.pone.0031718 (PMC3276513; doi:10.1371/journal.pone.0031718)
Supplement: Table S1 — Genes studied in the customized low density array. The genes of study are classified in groups depending on their role in muscle function: 1 = metabolism; 2 = membrane transport; 3 = mitochondrial function/biogenesis and dynamics; 4 = maintainance of cytoskeleton; 5 = contractile function; 6 = control of muscle growth; 7 = neuromuscular transmission; 8 = anti-stress protection; 9 = inflammation; 10 = cell death; 11 = calcium reuptake in sarcoplasmic reticulum; 12 = housekeeping gene. (DOC) [file pone.0031718.s001.doc]

**Supplementary Table 1**: Genes studied in the customized low density array. The genes of study are classified in groups depending on their role in muscle function: 1 = metabolism; 2 = membrane transport; 3 = mitochondrial function/biogenesis and dynamics; 4 = maintainance of cytoskeleton; 5 = contractile function; 6 = control of muscle growth; 7 = neuromuscular transmission; 8 = anti-stress protection; 9 = inflammation; 10 = cell death; 11 = calcium reuptake in sarcoplasmic reticulum; 12 = housekeeping gene.

| Gene Name | **Group** | **Gene symbol** | **TaqMan® Assay** | **Transcripts GenBank reference sequence** |
| --- | --- | --- | --- | --- |
| Acetil-CoA carboxylase beta | 1 | *ACACB* | Hs00153715_m1 | NM_001093 |
| Acetylcholine receptor, muscle, epsilon subunit | 7 | *ACHRE* | Hs00181084_m1 | NM_000080.2 |
| Acetil-CoA dehydrogenase medium chain | 1 | *ACADM* | Hs00163494_m1 | NM_000016 |
| Alpha-2 actinin | 5 | *ACTN2* | Hs00153809_m1 | NM_001103 |
| Alpha-3 actinin | 5 | *ACTN3* | Hs00153812_m1 | NM_001104 |
| Adenosine monophosphate deaminase I | 1 | *AMPD1* | Hs00163633_m1 | NM_000036 |
| Alpha keto acid dehidrogenase E1 subunit | 1 | *BCKDE1A* | Hs00167590_m1 | NM_000709 |
| Angiotensin I converting enzyme | 6 | *ACE1* | Hs00174179_m1 | NM_000789/ NM_152830 |
| ATP synthase 6, mitochondrial DNA encoded | 3 | *MT-ATP6* | Hs02596862_g1 | Chr. MT - 8528 - 9208 |
| Atrogin-1 | 6 | *ATROGIN* | Hs_00369714_m1 | NM_148177/ NM_058229 |
| Calcineurin | 6 | *CALNA* | Hs_00174223_m1 | NM_000944/ NM_001130691/ NM_001130692 |
| Calpain III large subunit | 4 | *CAPN3* | Hs_00544982_m1 | NM_173087/ NM_212464/ NM_212465/ NM_212467/ NM_024344/ NM_173088/ NM_173089/ NM_173090/ NM_000070 |
| Carnitine acyltranferase I | 1 | *CAT1* | Hs_00241357_m1 | NM_000755 / NM_004003 |
| Caspase 8 | 10 | *CASP8* | Hs_01018151_m1 | NM_033358/ NM_033355/ NM_033356/ NM_001080124/ NM_001080125/ NM_001228 |
| M-cadherine | 6 | *CDH15* | Hs_00170504_m1 | NM_004933 |
| Creatine kinase muscle type | 1 | *CKMM* | Hs_00176490_m1 | NM_001824 |
| Cytochrome b, mitochondrial DNA encoded | 3 | *MT-CYB* | Hs_02596867_s1 | Chr. MT - 14748 - 15882 |
| Cytochrome c | 3 | *CYCS* | Hs_01588974_g1 | NM_018947 |
| Cytochrome c oxidase subunit I, mitochondrial DNA encoded | 3 | *MT-CO1* | Hs_02596864_g1 | Chr. MT - 5905 - 7446 |
| Debranching enzyme | 1 | *GDE* | Hs_00240568_m1 | NM_000642/NM_000028/NM_000643/ NM_000644/NM_000645/NM_000646 |
| Desmin | 5 | *DES* | Hs_00157258_m1 | NM_001927 |
| Dinamin related protein 1 | 3 | *DRP1* | Hs_00247147_m1 | NM_012062/NM_012063/NM_005690 |

| Dystrophin | 5 | *DMD* | Hs_00187805_m1 | NM_004006/NM_000109/NM_004007/NM_004009/NM_004011/NM_004012/NM_004013/NM_004014/NM_004015/NM_004016/NM_004017/NM_004018/NM_004019/NM_004020/NM_004021/NM_004022/NM_004023/NM_004010 |
| --- | --- | --- | --- | --- |
| Glucose tranporter 4 | 2 | *GLUT4* | Hs_00168966_m1 | NM_001042 |
| Glycogen synthase muscle type | 1 | *GYS1* | Hs_00157863_m1 | NM_002103 |
| Heat shock 70 KD protein 2 | 8 | *HSPA2* | Hs_00356607_g1 | NM_021979 |
| Kinase phosphorylase 1 | 1 | *PHKG1* | Hs_00178864_m1 | NM_006213 |
| Monocyte chemotactic protein 1 | 9 | *MCP-1* | Hs_00234140_m1 | NM_002982 |
| Ring finger protein 28 | 6 | *RNF28* | Hs_00822397_m1 | NM_032588 |
| Myosin heavy chain 2 | 5 | *MYH2* | Hs_00430042_m1 | NM_001100112/ NM_017534 |
| Myostatin | 6 | *GDF8* | Hs_00193363_m1 | NM_005259 |
| Nebulin | 4 | *NEB* | Hs_00189880_m1 | NM_004543 |
| Nuclear respiratory factor 1 | 3 | *NRF1* | Hs_00602161_m1 | NM_005011/ NM_001040110 |
| Optic atrophy 1 | 3 | *OPA1* | Hs_01047018_m1 | NM_015560/NM_130831 |
| Muscle phosphofructokinase | 1 | *PFKM* | Hs_00175997_m1 | NM_000289 |
| PPAR-gamma coactivator 1 | 3 | *PPARGC1A* | Hs_00173304_m1 | NM_013261 |
| Sarcoplasmic reticulum Ca(2+)-ATPase 1 | 11 | *SERCA1* | Hs_00188877_m1 | NM_173201/ NM_004320 |
| Sarcoplasmic reticulum Ca(2+)-ATPase 2 | 11 | *SERCA2* | Hs_00544877_m1 | NM_170665/ NM_001681 |
| Superoxide dismutase 2 | 8 | *SOD2* | Hs_00167309_m1 | NM_001024465/ NM_001024466/ NM_000636 |
| Succinyl CoA transferase | 1 | *OXCT1* | Hs_00166467_m1 | NM_000436 |
| Troponin c, slow-twitch skeletal muscle | 5 | *TNNC1* | Hs_00268524_m1 | NM_003280 |
| Troponin c, fast skeletal | 5 | *TNNC2* | Hs_00268519_m1 | NM_003279 |
| Tumor necrosis factor alpha | 6 | *TNFA* | Hs_00174128_m1 | NM_000594 |
| Ubiquinol cytochrome c reductase core protein | 3 | *UQCRC1* | Hs_00163415_m1 | NM_003365 |
| 18s RNA | 12 | *18S* | Hs_99999901_s1 | X03205 |
| Large ribosomal protein | 12 | *RPLPO* | 4333761T | NM_053275 |
| Cyclophilin A | 12 | *PPIA* | Hs_99999904_m1 | NM_021130 |
| Transferrin receptor | 12 | *TRCF* | 4333770F | NM_003234 |
