# Supplementary material for: A Transcriptomic Approach to Search for Novel Phenotypic Regulators in McArdle Disease
Source: PLoS One. 2012 Feb 9;7(2):e31718. doi: 10.1371/journal.pone.0031718 (PMC3276513; doi:10.1371/journal.pone.0031718)
Supplement: Table S2 — Genes with statistically significant differential expression (p<0.05) between patients and controls. (DOC) [file pone.0031718.s002.doc]

| **Gene** | **Group** | **Mean (log_10_RQ)** | **Standard Desviation (log_10_RQ)** | **P<0.05** |
| --- | --- | --- | --- | --- |
| ACTN2 | Patients | -0.17 | 0.18 | 0.014 |
|  | Controls | 0.11 | 0.17 |  |
| BCKDE1A | Patients | -0.38 | 0.26 | 0.046 |
|  | Controls | -0.14 | 0.14 |  |
| MYH2 | Patients | -0.35 | 0.26 | 0.027 |
|  | Controls | -0.02 | 0.19 |  |
| OXTC1 | Patients | -0.62 | 0.33 | 0.046 |
|  | Controls | -0.31 | 0.20 |  |
| PFKM | Patients | -0.19 | 0.19 | 0.012 |
|  | Controls | 0.01 | 0.05 |  |
| PPARGC1A | Patients | -0.49 | 0.21 | 0.012 |
|  | Controls | 0.20 | 0.13 |  |
| SOD2 | Patients | -0.22 | 0.13 | 0.024 |
|  | Controls | -0.07 | 0.07 |  |
| TNNC1 | Patients | -0.20 | 0.15 | 0.019 |
|  | Controls | 0.02 | 0.13 |  |
| TNNC2 | Patients | -0.50 | 0.23 | 0.012 |
|  | Controls | -0.15 | 0.10 |  |
| UQCRC1 | Patients | -0.25 | 0.14 | 0.016 |
|  | Controls | -0.05 | 0.11 |  |

**Supplementary Table 2:** Genes with statistically significant differential expression (p<0.05) between patients and controls.
